# Statistical analysis plan for the Early Youth Engagement in first episode psychosis (EYE-2) study: a pragmatic cluster randomised controlled trial of implementation, effectiveness and cost-effectiveness of a team-based motivational engagement intervention to improve engagement

**DOI:** 10.1186/s13063-021-05670-2

**Published:** 2021-10-23

**Authors:** Christopher Iain Jones, Stephen Bremner, Richard Hooper, Jenny Gu, Gergely Bartl, Kathryn Greenwood

**Affiliations:** 1 Brighton and Sussex Medical School, Falmer, UK; 2 Institute of Population Health Sciences, Queen Mary University of London, London, UK; 3 School of Psychology, University of Sussex, Falmer, UK; 4 Research & Development, Sussex Partnership NHS Foundation Trust, Hove, UK

**Keywords:** Psychosis, Early intervention, Engagement, Intervention, Cluster randomised trial, Statistical analysis plan

## Abstract

**Background:**

Early Intervention in Psychosis (EIP) services improve health outcomes for young people with psychosis in the medium-long term, but 25% of young people disengage in the first 12 months with costs to their mental health, families, society and health services. This study will evaluate the effectiveness of a team-based motivational engagement intervention, the Early Youth Engagement (EYE-2) intervention.

**Methods and design:**

The EYE-2 trial is a cluster randomised controlled trial comparing the EYE-2 intervention plus standardised EIP service to standardised EIP service alone, with randomisation at the clinical team (cluster) level. The study aimed to enrol 950 young people (aged 14–35 years) with first episode psychosis in 10 teams per arm.

**Results:**

The primary outcome is time to disengagement: days from the date of allocation to care coordinator to date of the last contact following either refusal to engage with an EIP team or lack of response to EIP contact for 3 consecutive months which will be analysed using a shared frailty model. Secondary outcomes are Health of the Nation Outcome Scale (HoNOS), Process of Recovery Questionnaire (QPR), DIALOG (a service user-reported measure of quality of life and treatment satisfaction) and service use outcomes which will be analysed using mixed effects regression models.

**Discussion:**

This paper is the detailed statistical analysis plan for the EYE-2 trial. Any changes to, or deviations from, this plan will be described and justified in the final trial report.

**Trial registration:**

ISRCTN 51629746. Prospectively registered on 7 May 2019. Date assigned 10 May 2019.

**Supplementary Information:**

The online version contains supplementary material available at 10.1186/s13063-021-05670-2.

## Background

Early Intervention in Psychosis (EIP) services improve health outcomes for young people with severe mental illness in the medium-long term, but 25% of young people disengage in the first 12 months at significant cost to their mental health, their families, society and the National Health Service (NHS). Our own feasibility-pilot work clarified the issues that affect engagement. This study will refine and test the team-based motivational Early Youth Engagement (EYE-2) intervention to improve engagement and outcomes for young people as detailed in the published trial protocol paper [1]. The statistical analysis plan (SAP) was written following guidelines for SAPs by Gamble et al. [2].

### Study objectives

To evaluate the effectiveness of the intervention with respect to the primary outcome: time to disengagement (in days from date of allocation to care coordinator to date of the last contact following either refusal to engage with EIP or lack of response to EIP contact for 3 consecutive months) and secondary, routinely collected and researcher collected, outcomes (service use, deaths, Health of the Nation Outcome Scale (HoNOS) [3], Process of Recovery Questionnaire (QPR) [4], DIALOG—a service-user-reported measure of quality of life and treatment satisfaction [5]) derived from routine service data at 0, 6, 12, 18 and 24 months.

## Methods and design

### Trial design

EYE-2 is a cluster randomised controlled trial to compare the EYE-2 motivational engagement intervention plus standardised EIP service to standardised EIP service alone. Participating services are Early Intervention in Psychosis (EIP) clinical teams (henceforth referred to as teams) in 5 geographical locations in England including East Anglia (4 teams), Hampshire (4 teams), Manchester (5 teams), South London (3 teams) and Thames Valley (4 teams). These teams aim to promote recovery and reduce treatment delay for people experiencing first episode psychosis (FEP) by providing early access to multi-disciplinary support and treatment. Participating teams were expected to have at least 35–60 new FEP cases per year meeting participant inclusion criteria described below. A cluster randomised design was chosen to reduce the risk of contamination of the standardised EIP service teams with the EYE-2 intervention which is a team-based intervention. The intervention focuses on engagement, and although largely delivered by care coordinators (e.g. nurses, occupational therapists or social workers), it is supported by all members of the team including support workers, pharmacists, psychiatrists, psychologists and employment specialists.

Inclusion criteria for service users were defined as follows: (1) consecutive referrals to EIP services during the study recruitment period, (2) aged 14–35 years and (3) meeting criteria for a FEP as determined by each local service according to their own established criteria. Exclusion criteria were (1) sub-threshold ‘at risk mental state’, not meeting FEP criteria, (2) referrals over the age of 35, (3) diagnostic uncertainty about psychosis at 12 months and (4) service exclusion criteria such as organic or intoxication induced psychosis and specific exclusions.

The EYE-2 intervention [1] is a novel team-based motivational engagement model delivered by EIP teams of clinicians to young people with FEP. Key elements of the approach are (1) transparent, open communication; (2) social involvement and support from service users’ social network; (3) collaborative approach supporting client treatment choices; (4) hopeful supportive approach towards meaningful goals; and (5) addressing personal barriers to engagement. Clinicians delivering the approach are provided with training and an implementation toolkit consisting of an implementation manual, individual implementation checklists, online video resources and training, myth-busting booklets on (i) mental health and help-seeking, (ii) EIP services, (iii) advice for friends and family and (iv) treatment choices, and an engagement-focussed mental health companion website.

### Blinding

Teams administering interventions and individual participants are not blind to allocation. The research assistants rating the primary outcome are blind to the allocation status of teams at their respective sites. The statisticians, health economists and process evaluation research team members are also blind to allocation. The analysis will be conducted using dummy labels for the trial arms, using a code to which the statisticians are blinded. Final unblinding will only occur once the statistical analysis has been completed. SB had access to disaggregated data by coded treatment allocation for the purposes of separate exploratory work relating to the COVID-19 pandemic.

### Randomisation

A statistician at the Brighton and Sussex Clinical Trials Unit generated a randomisation list comprising permuted blocks of size 2, stratified by site using a tool provided by Sealed Envelope™ [6], an independent online randomisation service. To achieve statistician blinding and to ensure concealment, this was sent to an independent statistician to combine with the teams list, itself randomly ordered within site by sorting a random number list and then uploaded to Sealed Envelope. The study research fellow requested the password-protected concealed allocations online once all the participating teams at a site had reached the threshold for care coordinator and staff recruitment (≥ 80%) and were deemed ready to start.

### Power and sample size

Participants are a consecutive sample in each service. Time to disengagement will be analysed using frailty analysis to adjust for clustering by team. Simulation confirms that 10 clusters (teams) per arm (*N* = 950 participants in total across the 20 teams) will achieve 90% power to detect a difference corresponding to 12-month disengagement rates of 25% (standard 12-month disengagement rate from EIP service) [7–9] versus 15%, assuming time to disengagement follows an exponential distribution; intracluster correlation coefficient of 0.05 (to be conservative in the absence of information on this parameter); loss to follow-up rate 10% per year; conservative significance level 3% to correct for inflation of type I error due to the small number of teams; variable cluster size modelled as a uniform random variable between 35 and 60; recruitment at referral; 12 months’ recruitment plus 12 months’ follow-up. The target reduction in disengagement and expected loss to follow-up were based on data from the original EYE pilot project. We did not anticipate any loss of teams during the trial. Simulations were conducted using the SimSam package in Stata 14 [10], see https://github.com/richard-hooper/simsam/tree/EYE2.

In October 2019, the Trial Steering Committee asked us to re-evaluate the statistical power in light of the lower-than-expected rate of service user identification and to consider an extended timetable for the trial. We used recruitment figures to date to project the recruitment to each team if we recruited for a total of 15.5 months, and calculated power assuming an additional follow-up period of either 8.5 or 12 months. This re-calculation of power was done blind to the actual treatment allocations: simulations of power were conducted over all possible randomisations of teams stratified by site, with all other assumptions as in the original, pre-trial power calculation above. We estimated the power to be 85% with 15.5 months’ recruitment plus 8.5 months’ follow-up or 90% with 15.5 months’ recruitment plus 12 months’ follow-up.

### Statistical principles

#### Statistical software

Analyses will be performed in Stata version 16.1 or later [11].

#### Confidence intervals and *p* values

Estimates of treatment effect, their 95% confidence intervals and *p* values will be reported for comparisons between trial arms. The level of statistical significance is 5% (3% for the primary outcome).

#### Analysis population

Intention to treat principles will be followed to compare outcomes of the participants in each intervention arm.

#### Loss to follow-up

Participants will be considered lost to follow-up if they:

- Moved to a mental health service outside the study
- Moved to a service in a different arm of the trial
- Moved to a mental health service in a different country
- Died due to suicide
- Died due to other causes
- Asked to be withdrawn
- Withdrew for a safety reason
- Were discharged by mutual consent with no clinical need
- Other reasons—which will be stated

Engagement status of participants who are lost to follow-up will be censored at the time of their withdrawal.

Participants whose eligibility has been confirmed but their clinical team later revised their status as not FEP will be considered post-identification exclusions due to ineligibility and will not be included in the analysis. Participants who are rated blindly as disengaged will be followed up as per trial protocol.

### Timing of outcome assessments

The primary outcome is assessed on a 6 monthly basis by detailed case note review and trial database extracts, and indication of potential disengagement/loss to follow-up, by a research assistant blind to trial arm. Primary outcome status (engaged, disengaged, lost to follow-up) will be double-rated by an independent clinician based on all available data, and any discrepancies will be discussed to reach consensus. For those who have disengaged or have been lost to follow-up, the timing of this event will be retrospectively determined. For participants who remain engaged until the end of the study follow-up period, time to disengagement is treated as censored (unknown) beyond this point. This definition is widely used in engagement research. People who engage intermittently every few weeks or via text message or phone would still be engaged. Engagement will be evaluated according to the trial protocol and the EYE-2 Primary Outcome Rating Guidance Document (see Additional file 1) by double-rating, by raters blind to the allocation status of teams. Whilst final data entry and cleaning is conducted, we will continue to follow up engaged participants for a further 3 months to identify any final disengagement outcomes.

Secondary outcomes will be measured at 0, 6 and 12 months for all participants, not necessarily with each measure collected on the same date, and at 18 and 24 months for some, within an allowable window of −2 to +4 weeks either side of the due date, except for baseline for which the allowable window was −4 to +6 weeks and included the possibility that some data may have been collected whilst the participant was in an inpatient setting. Where more than one baseline value is available, we will select that which is closest to the date when the patient was allocated to their care coordinator. Due to the pressures of delivering clinical services, much of the secondary outcome data is collected outside the allowable windows. In order to accommodate this, such observations will be snapped to the nearest interim (pseudo) time point, i.e. 3, 9, 15 and 21 months.

### Adherence and protocol deviations

#### Definition of adherence to the intervention and extent of exposure

Adherence to the intervention will be measured at three different time points during the trial using self-report questionnaires administered to clinicians in each team as part of the process evaluation. Clinicians’ self-reported use of the key intervention resources ((i) EYE-2 booklets and (ii) website with service users and (iii) referrals of service users to social groups) will be used to assess the extent of exposure. For each of the three tangible components, clinicians rate the proportion of their EYE-2 service users to whom they have provided the intervention, rated on a 4-point Likert scale where 1 = 0–25% of my service users, 2 = 26–50% of my service users, 3 = 51–75% of my service users and 4 = 76–100% of my service users.

#### Presentation of adherence to the intervention

A composite mean adherence score will be calculated for each clinician by averaging their individual scores indicating the use of the three key intervention resources listed above with service users, providing a mean composite score ranging from 1 to 4. Summary statistics for adherence to the intervention will be reported for the intervention arm, and for each team, by averaging the scores for all clinicians in each team who completed the questionnaire at each of the three time points.

#### Protocol deviations

Data are collected at baseline and at 6 monthly regular follow-up intervals. Data can be collected within −4 to +6 weeks of baseline date (allocation to care coordinator) and within −2 to +4 weeks of follow-up time points. Data collected outside these periods will be classed as out-of-window data collection deviations.

The proportion of data collected out-of-window will be presented per trial arm for each time point for the following measures: HoNOS, QPR and DIALOG.

### Statistical analyses

#### Descriptive statistics

Participant progress through the study will be summarised using a flow diagram according to the CONSORT extension for cluster randomised trials [12], as presented in our trial protocol [1]. Screening data are collected by the research assistant for each team. We will report the number of patients screened, the number deemed ineligible (patients who did not meet team inclusion criteria of a new FEP presentation aged 14–35, at screening) with reasons and the number of eligible participants as a proportion of those screened.

Participant loss to follow-up will be summarised overall and by intervention arm in a table.

The following baseline characteristics will be summarised overall and by intervention arm:

- Age
- Ethnicity
- Gender
- Duration of untreated psychosis
- Level of educational attainment
- Deprivation score: Index of Multiple Deprivation [13]
- Substance use score on HoNOS at baseline
- Symptom score on HoNOS at baseline

Statistics appropriate to the distribution of each variable will be used, such as frequency and percentage for categorical variables, mean and standard deviation for normally distributed continuous variables or median and interquartile range for skewed continuous variables.

#### Interim analysis

No interim analysis was planned or conducted.

#### Outcome analyses

##### Primary outcome modelling: time to disengagement

Time to disengagement will be summarised by intervention arm and compared between trial arms using Cox regression with a gamma-distributed shared frailty to allow for the clustering by team. Missing values for the primary outcome, time to disengagement (due to loss to follow-up or remaining engaged at the end of data collection), are considered censored. The proportional hazards assumption will be assessed using Schoenfeld residuals. Should this assumption not be met, the model will be fitted after splitting the follow-up at the median (using Stata’s 'stsplit' command) and fitting the interaction between this and the randomisation variable. Alternatively, if the assumption is not met due to one or more covariates, we will stratify the modelling on these. If this analysis fails to converge, we will employ fully parametric time-to-event exponential regression analysis with a shared frailty. With a relatively small number of teams per arm, there is a risk that the type I error rate will be inflated—we will use a permutation test in order to obtain a true significance level [14]. Time to disengagement or the time beyond which observations are censored (due to loss to follow-up or end of data collection) will be known for all participants. Fixed effects will include treatment allocation, site, age at allocation to care coordinator and substance misuse score (HoNOS question 3) at baseline. We will report the 2-sided *p* value (and its 95% confidence interval) from the permutation test of the treatment effect. We will also report the estimated hazard ratio and its 95% confidence interval from the shared frailty model specified above.

##### Secondary outcomes

The following outcomes will be summarised by intervention and time point (up to 24 months) arm. Outcomes to be modelled are indicated with (m).

## 1. Service use

Service use data (primary endpoint 12 months), as advised by our GP commissioner, will include:

(i) Number of days spent in hospital (m)
(ii) Number of accident and emergency (A&E) presentations (m)
(iii) Number of instances of Section 136 use (m)

## 2. Health of the Nation Outcome Scale (HoNOS)

The HoNOS [3] is the most widely employed routine clinical outcome measure in the UK mental health services. It is a 12-item clinician-rated scale which covers a wide range of health and social outcomes including mental health symptoms (psychosis, depression, others), physical health, self-harm, substance use, cognition, function (occupational and daily), relationships and housing. Each item is rated from 0 (no problem) to 4 (very severe), for the preceding 2 weeks.

(i) Individual item scores
  We will model the hallucinations/delusions (m) item separately (question 6).
  Subscale scores
(ii) Behavioural problems (questions 1–3, range 0 to 12) (m)
(iii) Impairment (questions 4 and 5, range 0 to 8) (m)
(iv) Symptoms (questions 6–8, range 0 to 12) (m)
(v) Social (questions 9-12, range 0 to 16) (m)
(vi) Overall score (sum of 12 questions, range 0 to 48) (m)

## 3. Process of Recovery Questionnaire (QPR)

The QPR [4] is a 15-item measure, developed by psychosis service users to capture recovery. Items include social inclusion, assertiveness, motivation, positive relationships, purpose, empowerment, self-esteem, self-efficacy, meaningful activity, understanding, acceptance, enjoyment and positive risk-taking, each rated on a 5-point scale from 0, strongly disagree to 4, strongly agree. The outcome is the total score for all 15 items.

(i) Overall score (sum of 15 questions, range 0 to 60) (m)

## 4. DIALOG

The DIALOG [5] assesses patient-reported satisfaction with 11 aspects of subjective quality of life including health (mental and physical), function (work, leisure), social (friendships/family relationships), accommodation, personal safety and treatment (practical and mental health support, medication) all rated on a 7-point scale from 1, totally dissatisfied, to 7, totally satisfied. Scores on the DIALOG are reported as two mean subscale scores for Subjective Quality of life and Satisfaction with treatment.

Subscale scores

(i) Subjective quality of life (mean of questions 1-8, range 1 to 7) (m)
(ii) Treatment satisfaction (mean of questions 9-11, range 1 to 7) (m)

## 5. Death

Death, including suicide, within 12 months of allocation to care coordinator.

## 6. National Institute of Health and Care Excellence (NICE) guideline interventions

Number of NICE guideline interventions within 12 months.

### Secondary outcome modelling

As not all participants are followed up beyond 12 months, the greatest effort has been focused on collecting data to 12 months; therefore, this is the data that will be used for the modelling. HoNOS, QPR and DIALOG will be analysed using mixed effects regression analysis of all non-missing data (valid if outcomes are missing-at-random) appropriate to distribution, with random effects for time point and for team and a Kenward-Roger small-sample correction to account for the small number of teams. From each model, we will report the estimated treatment effect, its *95% CI* and *p* value. If any of the models do not converge, we will report a descriptive comparison of the outcomes by trial group. Service use outcomes will be modelled using mixed effects robust Poisson regression with a random effect for team to account for clustering at the team level, and the fixed effects listed below. We will report the estimated incidence rate ratio for the treatment effect and its *95% CI*. Due to the small number of teams, a permutation test will be used to calculate the *p* values.

Models will include the following fixed effects:

- Site
- Treatment allocation
- Outcome at baseline (HoNOS only)
- Age at allocation to care coordinator
- Time point

A substantial number of observations for HoNOS, QPR and DIALOG fall outside the collection windows for each time point. Observations for these outcomes falling outside expected collection windows will be quantified and visualised. Intermediate (pseudo) time points at m3 and m9 will be created between the main time points (m0, m6 and m12), and observations will be assigned to the closest empty time point. For HoNOS, baseline observations will not be reassigned to later time points as data collection for HoNOS occurred close to baseline, and baseline score will be included as a covariate in the model. For QPR and DIALOG, baseline data collection could occur after true baseline, so these observations may be reassigned to their closest empty time point; otherwise, the baseline score will instead be included in the outcome variable. An interaction between treatment allocation and time will be included in these models, but the treatment allocation (intervention) main effect will not be included (QPR and DIALOG models), as this represents a comparison of the outcome at baseline. Omission of the treatment allocation main effect is equivalent to assuming equality of outcome between groups at baseline [15].

### Missing data for secondary outcomes

The specified analysis of HoNOS, QPR and DIALOG is based in the assumption that unobserved participants are missing-at-random (MAR). For each of these outcomes, we will examine the sensitivity of conclusions to this by imputing outcome data departing from this assumption [16-19]. This will be performed using Δ = Δoutcome + Y_1_P_1_ – Y_0_P_0_, where Δ is the treatment effect under MNAR, Δoutcome is the treatment effect, estimated with the ‘margins’ command in Stata, from each secondary analysis model, Y1_1_ and Y_0_ are the assumed mean participant responses of those missing data in the ‘EYE-2 intervention plus standardised EIP service’ and ‘standardised EIP service alone’ arms respectively, and P_1_ and P_0_ are the proportions of participants excluded from analysis in their respective arms. Y_0_ will be varied for each outcome variable, and for each value of Y_0_, Y_1_ will be set equal to: {Y_0_ - x, Y_0_, Y_0_ + x}. The assumed mean response (Y_0_) of participants missing data in the ‘standardised EIP service alone’ arm will be varied across the following values: HoNOS: {5, 10, 15, 20, 25, 30, 35, 40, 43}; QPR: {10, 20, 30, 40, 50}; and DIALOG: each subscale {2, 3, 4, 5, 6}. The assumed mean response (Y_1_) of participants missing data in the ‘EYE-2 intervention plus standardised EIP service’ arm will be set equal to Y0_0_ + x for each x as follows: HoNOS: {-5, 0, 5}; QPR: {-10, 0, 10}; DIALOG: {-1, 0, 1}. We will calculate confidence intervals for the treatment effect using the standard error of Δoutcome, assumed approximately equal to the standard error of Δ, from the complete case analysis. An example of the application of this approach can be found in Gillard et al. [20].

### Additional analyses

#### Sensitivity analyses

- HoNOS, QPR and DIALOG models including only observations collected in window.
- Method of data collection for HoNOS: We will explore the impact of different methods of collection of HoNOS, namely whether there are differences between outcomes collected by care coordinators and those collected by research assistants via case notes screen (with/without checking by care coordinator) or telephone interview with the participant, by fitting a model which includes only participants whose data were collected by their care coordinator.
- Impact of the COVID-19 pandemic: We will use the date of the first UK lockdown (16/3/21) and fit two models for each of disengagement, HoNOS, QPR and DIALOG outcomes up to 12 months (1) including only participants with baseline measure ascertained before lockdown and (2) including only participants with baseline data collected after lockdown and pool the estimates from each pair of models using a fixed-effects meta-analysis.

#### Subgroup analyses

The primary analysis model will be repeated on the following subgroups (in separate models) by fitting an interaction term between the subgroup variable at baseline and the treatment allocation variable as follows:

- Substance misuse—binary 2, 3 or 4 vs. 0 or 1 on HoNOS item 3
- Symptom severity (HoNOS symptom score)
- Ethnic group (mixed/other, any Black, any Asian vs. any White)
- Educational attainment (degree, vocational, A-level, GCSE vs. none)
- Deprivation category (high vs. low)
- Sex (male vs. female only)
  Additionally for disengagement and HoNOS, QPR and DIALOG, using the research assistant questionnaire or EIP triangulation tool:
- Average caseload per care coordinator in the team (high vs. low)
- Funding level per team (high vs. low)

### Harms

Serious adverse events possibly, probably or definitely related to the study intervention, will be summarised by intervention arm and overall. This will include counts of any events which resulted in death, were life-threatening, required new or prolonged hospitalisation, resulted in persistent or significant disability/incapacity, or any other medically significant event.

## Trial status

Follow-up for the trial finished on 6 July 2021 which is 12 months after the last participant was recruited. The statistical analysis is anticipated to commence in October 2021 following a period of final data entry, data cleaning and data lock.

## Supplementary Information

**Additional file 1:.** EYE-2 Primary outcome rating guidance document

## Data Availability

An anonymised version of the main outcome data will be available from the corresponding author on reasonable request after publication of the main results paper.

## Abbreviations

CAARMS: Comprehensive Assessment of At Risk Mental States
EIP: Early Intervention in Psychosis
EYE: Early Youth Engagement
HoNOS: Health of the Nation Outcome Scale
MAR: Missing-at-random
MNAR: Missing-not-at-random
NHS: National Health Service
NICE: National Institute for Health and Care Excellence
PANSS: Positive and Negative Symptoms Scale
QPR: Process of Recovery Questionnaire
RCT: Randomised controlled trial
