# Supplementary material for: Statistical analysis plan for the Early Youth Engagement in first episode psychosis (EYE-2) study: a pragmatic cluster randomised controlled trial of implementation, effectiveness and cost-effectiveness of a team-based motivational engagement intervention to improve engagement
Source: Trials. 2021 Oct 23;22:732. doi: 10.1186/s13063-021-05670-2 (PMC8541800; doi:10.1186/s13063-021-05670-2)
Supplement: Supplementary file 1 — Additional file 1:. EYE-2 Primary outcome rating guidance document [file 13063_2021_5670_MOESM1_ESM.docx]

**EYE-2 Primary outcome rating guidance document**

Contents

[Useful contacts 1](#_Toc76121592)

[Background 1](#_Toc76121593)

[Key terms 2](#_Toc76121594)

[Engagement/disengagement rating process 4](#_Toc76121595)

[Research Assistants 4](#_Toc76121596)

[The role of the un-blinded Research Assistant (ongoing). 4](#_Toc76121597)

[The role of the blinded Research Assistant (ongoing). 5](#_Toc76121598)

[Blinded RA cover. 5](#_Toc76121599)

[Engagement status review at the end of trial follow-up period (6^th^ July 2021) 5](#_Toc76121600)

[Participants with uncertain engagement status at the end of study follow-up period 6](#_Toc76121601)

[Engagement rating by a second blind rater (July to Oct 2021) 7](#_Toc76121602)

[Appendices 8](#_Toc76121603)

[Appendix 1 – Engagement Status Form (MACRO - https://macro.infermed.com/bsctu/) 8](#_Toc76121604)

[Appendix 2 – Engagement rating at follow-up time points (6, 12, 18 months) 9](#_Toc76121605)

[Appendix 3 – Engagement rating when participant may be lost to follow-up or reaches end date of trial (6^th^ July 2021) 10](#_Toc76121606)

[Appendix 4 – Additional blind rating of engagement status (July to Oct 2021) 11](#_Toc76121607)

# Useful contacts

The Research Fellows are available to answer any queries relating to this guide: contact [research fellow] if you have any questions at any time.

# Background

The EYE-2 cluster randomised controlled trial evaluates the effectiveness of a team-based motivational Early Youth Engagement (EYE) intervention and toolkit in Early Intervention in Psychosis (EIP) services. This is done by comparing service users’ engagement and other outcomes between two arms of the study: (1) standardised EIP services and (2) standardised EIP services with additional EYE-2 intervention. The primary outcome is participants’ time to disengagement, defined as the time in days from date of allocation to care coordinator to date of last contact with the service, following either (a) refusal to engage with EIP service, or (b) lack of response to EIP contact for a consecutive 3-month period. This guidance document details the process, key terms, roles, and timeframe involved. Un-blinded and blinded Research Assistants (RA) and additional reviewers contribute to this process during the trial. This document should be read in the context of the current EYE-2 study protocol and the Glossary of terms document.

# Key terms

Time to Disengagement: Time in days from date of allocation to care coordinator (as recorded on the MACRO database, see Appendix 1) to the date of last contact when a service user refused to engage with the EIP service or after which there has been a lack of response to EIP contact for a consecutive 3-month period.

Disengagement: Engagement should normally be rated at 6 months intervals following allocation to care coordinator, and at the end of the study. A person should be rated as ‘Disengaged’ at the reviewed time point if they either (1) refused to engage with EIP service, or (2) they did not respond to EIP contact for a consecutive 3-month period. If a service user has been rated disengaged at a time point, their primary engagement rating should remain ‘disengaged’ at all subsequent time points. Note that if the above disengagement criteria are not met, participants should be rated as ‘Engaged’ for that time point. Additional criteria are also included below to further clarify the distinction between disengagement and being lost to follow-up.

Forms of disengagement:

1. Refusal to engage with the EIP team: This disengagement reason covers instances when a service user is no longer in contact with the EIP team following explicitly refusing contact with or input from the EIP team. Participants meeting this criterion should be rated as ‘Disengaged’.
2. Lack of response to EIP contact for 3 consecutive months: if following allocation to a care coordinator an EIP team unsuccessfully tried to make contact with a service user (with no reply from the client) for a 3 month period, the participant should be rated as ‘Disengaged’.
3. Service user leaves the area or the country with no on-going transfer of care, or refusal of ongoing transfer of care, despite this being available and despite therapeutic need.
4. Other reason, e.g. Request to stop receiving EIP service support despite therapeutic need, with or without the team’s agreement. This encompasses situations where a service user says they no longer want EIP support or asks to stop receiving support, within 2 years of first allocation and despite offers of ongoing or alternative support from the team or signs of on-going need. This may be rated as disengaged even if the team ultimately agree to the discharge.

Contact: these are in-person meetings (attended), phone calls (answered), text messages and emails with the service user present to which the service user actively responded (replying in email, text or voicemail message- note if the response is only to restate a desire not to be seen this does not count as a contact). Note that meetings or calls by the EIP team with family members/friends present but not the service user themselves should not be counted as attended. Similarly, unsuccessful attempts by the team to call/meet the service user should not be rated as attended or responded to by the service user.

Date of last contact: this is the last known recorded clinical contact with the service user at the time of the engagement rating. E.g. this could be the day of the last appointment they attended in person, the day of the last phone call they made to the team or responded to, or the date when the last emails/texts were sent to the team by the service user.

Frequency of engagement ratings: Engagement should be reviewed on a 6-monthly basis throughout the trial, i.e. at 6, 12, and 18-months follow-up times. In addition, in order to determine the engagement status of all participants at the end of their trial participation, all participants’ engagement rating will be reviewed at respective last day, i.e. the last day of the trial (6^th^ July) or the day of their loss to follow-up (but see below for details of lost to follow-up definition). Note: from the trial’s perspective, disengagement is a one-off event: participants rated as ‘Disengaged’ at any point during the trial should remain rated disengaged at all subsequent time points.

Lost to follow-up: Participants will be considered lost to follow-up if (i) they move to a mental health service outside the study or (ii) they move to a service that is in a different arm of the EYE project or they move out of the UK with no mention of disengagement and cannot be referred to a mental health service. These participants will no longer be receiving the intervention and are deemed lost to follow-up. The key distinction here between someone who is lost to follow-up vs someone who is disengaged is that someone who is lost to follow-up has moved and actually taken up another service, so they have remained engaged but they are now no longer receiving treatment with one of the study teams so their subsequent data is no longer relevant OR they have moved to a country that does not have an identifiable mental health service to which they can be referred, so they are also considered to be lost to follow-up. NOTE: data for participants who are lost to follow-up will be used up to the point at which they move. Please note the following key concepts:

1. Managed transfer of care: Service users may re-locate to a different area in agreement with the team and receive appropriate follow-up and engage in a discharge plan. However, instances when moving out of the EIP team’s area to a new location or different country is against clinical advice, and without a managed transfer of care this should be considered as disengagement.
2. Mutually agreed discharge as service user is well/fully recovered and the team has nothing to offer: Service users may leave the team because they have recovered and are well and the team discharge them with the service users’ agreement. In which case this would also be a loss to follow-up as they are no longer receiving a service; BUT if there is any sign that this was at the service users request and that the team could/would have continued to support them clinically otherwise then this should be classed as disengagement.
3. Moved to a country with no identifiable mental health service and so could not be referred to a new service.

Participants lost to follow-up are recorded on MACRO database as one of the categories below:

1. Participant asked to be withdrawn from the research study
2. Moved to a mental health service outside the study and cannot be followed up
3. Moved to a service that is in a different arm of the EYE project (data will be used up to this point
4. Safety reason (specify)
5. Death due to suicide
6. Death due to other cause (specify)
7. Mutually agreed discharge (recovered/no longer needs service)
8. Post identification exclusion (not meeting eligibility criteria)
9. Moved to a country with no identifiable mental health service
10. Other reason

The rating of engaged, disengaged, or lost to follow-up comprise the primary outcome of the study so it is critical that this rating is correct. If you are in any doubt about this rating at all, and for any queries not covered in this guide – please discuss this immediately with the Research Fellows and site PIs on a case-by-case basis providing all relevant data before recording on MACRO.

# Engagement/disengagement rating process

## Research Assistants

(See also Appendix 2 and 3 flowcharts)

### The role of the un-blinded Research Assistant (ongoing).

The un-blinded RA will monitor spreadsheets and clinical notes / team meetings on a weekly basis in order to identify service users reaching the 6, 12, or 18-month time point, and service users for whom there is a query of being lost to follow-up. For participants meeting any of the above criteria, the un-blind RA will carry out a detailed case note screen and make available all information that the blinded RA colleague will need to make a clear rating via the MACRO Engagement Status form (Appendix 1). This process is **ongoing** and involves the following steps:

- Review clinical records since baseline or the last engagement review, whichever more recent.
- Write a set of detailed and verbatim notes on recent contacts and appointments attended or scheduled between the service user and the EIP team.
- In these, particularly include: the engagement-review due date (e.g. *‘6m follow-up due 14/2/2021’*), list of recent appointments with corresponding date and brief content (*e.g. ‘12/2/2021: Meeting between patient and Care Coordinator, discussed benefit application’*)
- Make a note if recent appointments were not attended by the participant (e.g. *(‘10/1/2021: Did not attend CPA’; ‘17/1/2021: Tried to contact participant, no response’; 22/1/2021: Home visit, only parent attended, service user DNA’*)
- Include any information which may indicate that the service user disengaged or is disengaging. E.g. *(‘12/3/2021: Service user left voicemail to request not to be contacted by the team again’*).
- Include any information which may indicate whether the service user is leaving the service/being discharged/has moved to a new location against clinical advice.
- Include any information which may indicate whether the service user is leaving the service/being discharged/has moved to a new location with agreement of the team, and/or with a managed transfer of care.
- Include any information that suggests offers of ongoing support from the team or evidence of ongoing therapeutic need.
- Include any information that suggests a prior pattern of disengagement, expressions of desire to disengage or stop receiving support or family encouragement to disengage which the service user agrees with
- Upload the completed case note screen notes to MACRO by right-clicking on the ‘Comment added’ box (see Appendix 1). Tick the box to indicate the upload is complete.
- Record engagement rating in a spreadsheet, but do not disclose this to blinded RA colleagues.
- Inform their blind RA counterparts that case notes ready to be rated.
- Update the case note screen on MACRO if further information is requested by the blinded RA to enable them to complete the rating.

### The role of the blinded Research Assistant (ongoing).

The blinded RA will review case notes uploaded to the MACRO Engagement Status form (Appendix 1), at each time point (6, 12, 18 months, end of study) or if there is suspicion of disengagement or being lost to follow-up, match the information against the trial engagement, disengagement and lost to follow-up criteria above, and record engagement status on the form for each trial participant. This process is **ongoing** and involves the following steps:

- Become familiar with disengagement and lost to follow-up criteria in the current protocol and review the ‘Key terms’ relating to engagement status/disengagement defined above in this document.
- Review case notes on MACRO by right clicking on the ‘Comments added’ box on the Engagement status form.
- Record the service user’s engagement status in the relevant ‘Current status’ field:
  - If the service user meets the disengagement criteria (see ‘Key terms’), record them as ‘Disengaged’.
  - If there is not sufficient information, request more information from the un-blinded RA and re-review later.
  - If the disengagement criteria were not met, record the service user as ‘Engaged with the service’.
  - If the service user meets criteria for lost to follow-up, then record ‘Withdrawn’ in the separate MACRO section ‘Ongoing -> Withdrawals’.
- Record ‘Date of last contact’ (see ‘Key terms’).

### Blinded RA cover.

In sites where there is a separate blinded RA within the team, the above tasks are split between the un-blinded and blinded RA of the sites. Sites where there are not any blinded RAs employed directly tasks are shared with one other site.

## Engagement status review at the end of trial follow-up period (6^th^ July 2021)

For all participants, engagement status is monitored at 6-monthly intervals until the end of the study follow-up period or disengagement/becoming lost to follow-up, whichever is sooner. For all service users not yet rated as disengaged or lost to follow-up by the 6^th^ July, a final review of engagement status will be carried out:

- The unblinded RA will screen case notes up to 6^th^ July and record them on MACRO.
- The blinded RA will rate participants as per protocol:
  - Disengaged: rate participant, record Date of Last Contact. End of follow-up.
  - Lost to follow-up (Withdrawn): rate participant on MACRO, record Date of Last Contact. End of follow-up.
  - Engaged with recent Date of Last Contact (on or after 22^nd^ June 2021) – Rate participant on MACRO, record Date of Last Contact. End of follow-up.
  - Engaged with Date of Last Contact earlier than 22^nd^ June 2021: Rate participant on MACRO, record Date of Last Contact. Note participants’ MACRO ID for later review as described below in *‘Participants with uncertain engagement status at the end of study follow-up period’*.

## Participants with uncertain engagement status at the end of study follow-up period

Service users may meet the disengagement criteria if they do not respond to EIP contacts for a three-month period. Therefore, case notes of participants with a date of last contact within the last three months of the trial follow-up period will be evaluated additionally for disengagement up to three months after the trial end date (6^th^ Oct 2021) as follows:

- The blinded RA and Research Fellow, together with the CTU/MACRO team will maintain a list of service users per site who were rated as ‘Engaged with Date of Last Contact earlier than 22^nd^ June 2021’ (as within a fortnight of trial end) by the blinded RA.
- Unblinded Research Assistants will review and upload clinical case notes regarding engagement status for this period and upload on MACRO – as soon as one contact has occurred beyond 22^nd^ June this person will be counted as ‘Engaged’ at trial end. End of follow-up.
- Blinded RAs will review the engagement status of participants from the list and update engagement/disengagement information on MACRO as appropriate:
  - All participants who have attended a further contact with the EIP team after 22 June will remain rated as ‘Engaged’.
  - Participants who have met the disengagement criteria (no response to EIP contact) in the 3 months since Date of Last Contact (pre 22^nd^ June) as recorded on MACRO will be rated as ‘Disengaged’.

## Engagement rating by a second blind rater (July to Oct 2021)

(See also Appendix 4. flowchart)

In order to evaluate the reliability of ratings at the end of the trial, service users’ engagement will also be reviewed by a second clinical blind rater applying the same trial protocol criteria. After the end of the follow-up period (6^th^ July 2021) a second rater with clinical experience in EIP / psychosis services will be provided engagement status case notes. This will be used to assess the reliability and sensitivity of ratings. This process is planned in **July to Oct 2021** and involves the following steps:

- The Research Fellow will provide case notes from MACRO to the second blind clinician rater in an anonymous format in a password-protected document, with the initial engagement rating of the blind RA removed.
- The second blind clinical rater will rate engagement/disengagement/lost to follow-up according to study protocol, following the steps described above under ‘The role of the blinded Research Assistant’.
- The second blind rater will liaise with the Research Fellow and the Research Assistants if any further clinical information is needed for their decision.
- The Research Fellow will review the concordance rate between the two ratings to establish inter-rater reliability.
- Disagreements between the ratings made by the blind RA and the second blind rater will be reviewed by a third blind rater who will resolve conflicts and finalise blind rating of primary outcome before data analysis.

# Re-engagement rating process

Re-engagement: This is an extra field on the MACRO Engagement status form (Appendix 1) that has been added for people who have disengaged. This is to capture any instances where someone re-engages with the service again after disengaging. This should be done for all participants who have previously disengaged from the service at any point during the trial. Un-blinded RAs should check case notes at the end of the trial to evaluate whether re-engagement occurred. I.e. for all participants rated as disengaged, and they can in addition be rated as re-engagement by community contact or inpatient admission if this happens DO NOT CHANGE THEIR DISENGAGEMENT STATUS. This process is planned in **July 2021** and involves the following steps:

- The un-blinded RAs and the Research Fellow will compile a list of service users at the site who were rated as disengaged.
- The un-blinded RA will review clinical records to note whether re-engagement occurred at any point following disengagement.
- The un-blinded RA will complete the Re-engagement section on MACRO (Appendix 1).

# Appendices

## Appendix 1 – Engagement Status Form (MACRO - https://macro.infermed.com/bsctu/)


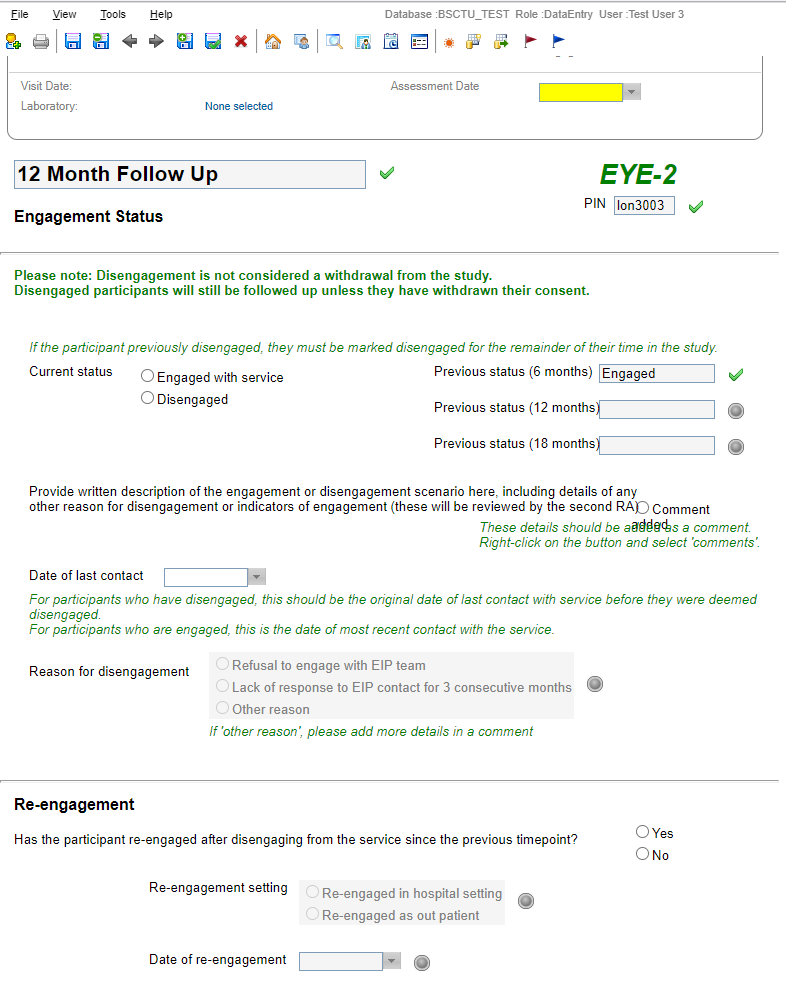


## Appendix 2 – Engagement rating at follow-up time points (6, 12, 18 months)

1. Participant reaches a follow-up time point
2. Un-blinded RA carries out case note screen
3. blinded RA to rate, outcome:

- a: meets disengaged criteria
- b: engaged
- c: more info needed – contact the Un-blinded RA

1. Additionally, blinded RA to complete withdrawal form on MACRO if applicable

## Appendix 3 – Engagement rating when participant may be lost to follow-up or reaches end date of trial (6^th^ July 2021)

1. Participant reaches end of study (6^th^ July) OR Un-blinded RA identifies potential lost to follow-up
2. Un-blinded RA carries out case note screen
3. Blinded RA to rate outcome:

- a: meets disengaged criteria
- b: engaged
- c: more info needed

1. Additionally, blinded RA to complete form on MACRO if applicable
2. Follow up:

- if disengaged: continue to follow as trial participant
- if meets lost to follow-up criteria: mark participant as lost to follow-up participant on MACRO

## Appendix 4 – Additional blind rating of engagement status (July to Oct 2021)

1. After the end of the follow-up period (6^th^ July 2021) the Research Fellow compiles anonymous case notes from MACRO (original engagement rating not included), for all disengaged/lost to follow-up cases, and cases still engaged. The research fellow sends this to the 2^nd^ blind rater in an anonymous, password protected file.
2. 2^nd^ blind rater reviews as per protocol and guidance document and records ratings in an excel file template and returns this to the research fellow.
3. The Research Fellow reviews data for agreement/disagreement and provides information regarding disagreements to a third blind rater in an anonymous, password protected document.
4. 3^rd^ blind rater reviews and resolves disagreements.
5. All cases rated as ‘uncertain engagement status’ based on data at 6^th^ July to be re-reviewed once additional data becomes available (either a new contact or 3 months of no-contact) following the same protocol as outlined above.
6. Finalised engagement status data (including resolved conflicts between the blind RA and second blind rater) to be included in statistical analysis of primary outcome.
